# Pleiotropic Role of Puupehenones in Biomedical Research

**DOI:** 10.3390/md15100325

**Published:** 2017-10-21

**Authors:** Beatriz Martínez-Poveda, Ana R. Quesada, Miguel Ángel Medina

**Affiliations:** 1 Department of Molecular Biology and Biochemistry, Faculty of Sciences, University of Málaga, Andalucía Tech, and IBIMA; E-29071 Málaga, Spain; bmpoveda@uma.es (B.M.-P); quesada@uma.es (A.R.Q.); 2 Unidad 741 de CIBER “de Enfermedades Raras”, E-29071 Málaga, Spain

**Keywords:** puupehenones, sponges, marine drugs, antiangiogenic, antitumoral

## Abstract

Marine sponges represent a vast source of metabolites with very interesting potential biomedical applications. Puupehenones are sesquiterpene quinones isolated from sponges of the orders *Verongida* and *Dictyoceratida*. This family of chemical compounds is composed of a high number of metabolites, including puupehenone, the most characteristic compound of the family. Chemical synthesis of puupehenone has been reached by different routes, and the special chemical reactivity of this molecule has allowed the synthesis of many puupehenone-derived compounds. The biological activities of puupehenones are very diverse, including antiangiogenic, antitumoral, antioxidant, antimicrobial, immunomodulatory and antiatherosclerotic effects. Despite the very important roles described for puupehenones concerning different pathologies, the exact mechanism of action of these compounds and the putative therapeutic effects in vivo remain to be elucidated. This review offers an updated and global view about the biology of puupehenones and their therapeutic possibilities in human diseases such as cancer.

## 1. Origin and Biological Role of Puupehenones in Sponges

The need of new pharmacological approaches for the treatment of certain refractory diseases is the starting point for a growing research field in recent years. Indeed, discovery and characterization of new natural products and derivatives with potential therapeutic activity are key issues in pharmacological research. Natural products under investigation have a wide variety of origins, but some of the most important sources of candidate compounds for biomedical applications are marine organisms. Among the great biodiversity present in oceans and seas, sponges represent a real treasure for the isolation of new compounds with unique structural characteristics, due to the synthesis in these organisms of a high number of secondary metabolites. Sponges (phylum *Porifera*) are sessile and filter-feeder multicellular organisms that lack body symmetry. The soft body of the majority of sponges and the incapacity of movement make these organisms a perfect target for predators (fish, turtles and invertebrates); the adaptive strategy of sponges to such threats is the synthesis of chemical compounds that have a defensive role to deter predators [1]. The chemical nature of these compounds is very diverse, including sterols, terpenes, cyclic peptides, alkaloids, fatty acids, peroxides, amino acid derivatives (frequently halogenated) and unusual nucleosides [2].

One group of marine compounds synthesized by sponges that deserves special attention is the group of the puupehenones. Puupehenones are shikimate-derived sesquiterpene quinones whose isolation has been reported mainly from the orders *Verongida* and *Dictyoceratida*, although some compounds from this family have been identified as well in orders *Dendroceratida* and *Haplosclerida* [3]. Among all the compounds that belong to this family, puupehenone is the most representative member. It was firstly isolated and described by B. N. Ravi and colleagues in 1979, who named the compound in honor of the legendary Hawaiian princess Puupehe [4], but its absolute stereochemistry was not elucidated until 1996 [5].

Puupehenone and other related compounds exhibit very potent cytotoxic and antimicrobial activities, pointing to their possible role as defensive weapons in sponges. Apart from these detected activities that could be important in the chemical ecology of sponges, the exact role of puupehenones in sponges’ biology is not fully defined, although an interesting mechanism has been proposed for puupehenone by which this metabolite could participate in the detoxification of excess of hydrogen cyanide (HCN), probably produced by sponges as chemical weapon with defensive purpose [6]. It has been reported that harvested sponges from the order *Verongida* emitted HCN when they were broken apart, and this observation correlates with the necessity of a mechanism of detoxification of this toxic compound in the sponge [7]. The easy conversion of puupehenone into its cyano-derivatives (15α-cyanopuupehenol and its oxidation product 15α-cyanopuupehenone) by the addition of hydrogen cyanide under aqueous conditions suggests a possible hydrogen–cyanide–puupehenone cycle, highlighting the putative biological function of puupehenone in the sponge’s biochemical system [6].

Our group has contributed to the knowledge of puupehenones, focusing on their activity as antiangiogenic and pro-apoptotic compounds [8,9]. Their potential as antitumoral compounds makes puupehenones a very interesting family of metabolites for biomedical and pharmaceutical research. The information compiled in this review tries to provide an updated and global view about puupehenones’ biology and their therapeutic possibilities.

## 2. Diversity and Chemical Synthesis of Puupehenones

**Figure 1:**
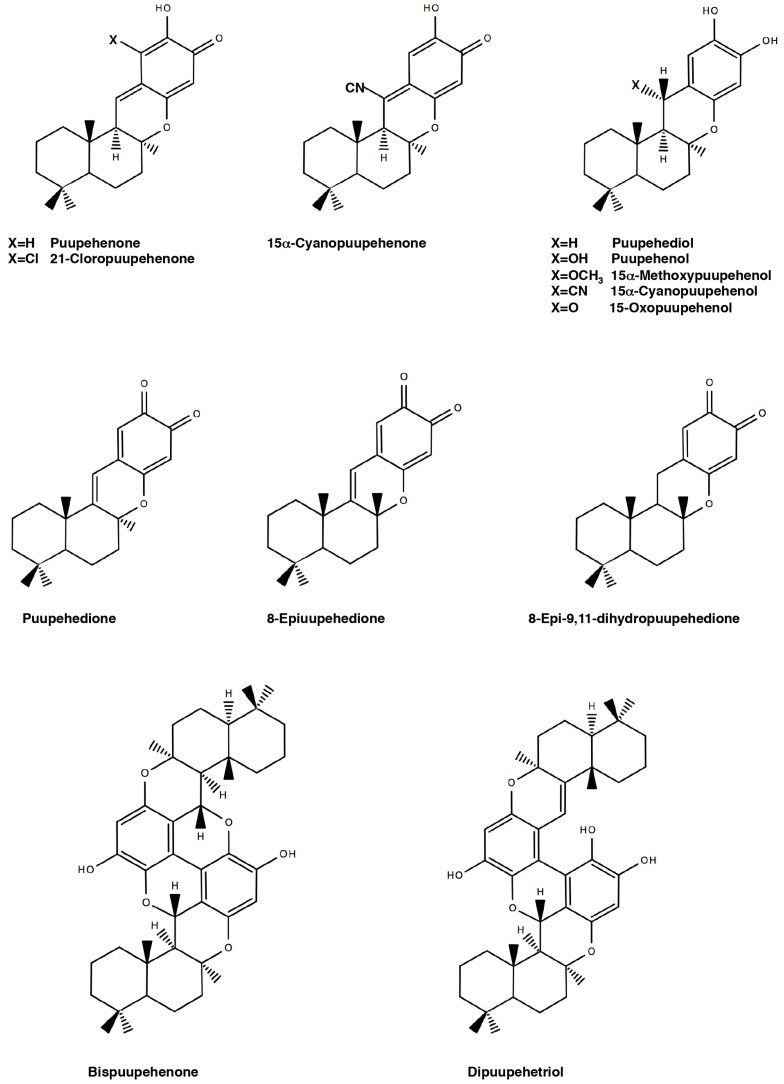
Chemical structure of puupehenone and some derived compounds.

The compounds gathered in the family of puupehenones are very diverse (Figure 1) and chemically belong to the large group of the sesquiterpene quinones. They have very characteristic structures, presenting a common tetracyclic core (a sesquiterpene unit joined to a phenolic moiety). Puupehenone, the most representative compound of this family, structurally differs from other sesquiterpene quinones because of the presence of a quinone–methide system responsible for its unique chemical behavior; it exhibits high chemical reactivity, facilitating the formation of many derived metabolites. The 1,6-Conjugated nucleophilic addition of HCN to puupehenone in the presence of water and alkaline conditions yields 15α-cyanopuupehenol and its oxidation product 15α-cyanopuupehenone [6]. Addition of oxygen nucleophiles such as acetoxy and methoxy ions to puupehenone (obtaining 15α-acetoxypuupehenol diacetate and 15α-methoxypuupehenol) has been also reported [4,10]. A large number of puupehenone-derived/related compounds, either naturally occurring or of synthetic origin, has been reported in the literature [11]. Some of them are shown in Figure 1.

**Figure 2:**
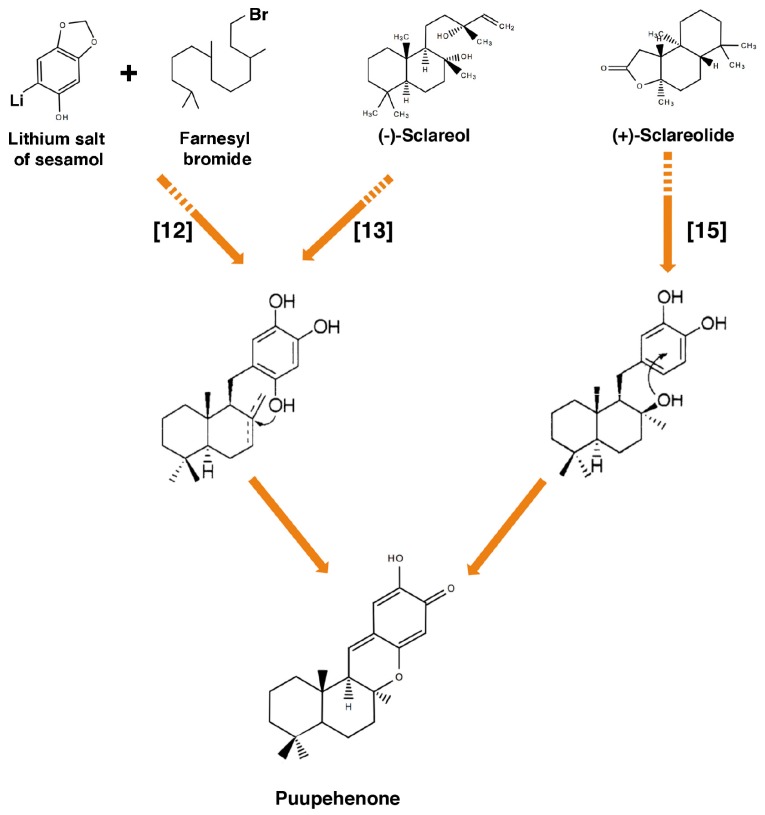
Chemical strategies for the synthesis of puupehenone. Scheme of the synthetic strategies reported in [12,13,15] for the synthesis of puupehenone, showing the starting compounds and the differential steps used to obtain the heterocyclic oxygen in each approach. Adapted with permission from [15].

Chemical synthesis of several puupehenones has been reported, using different synthesis routes and several initial compounds (Figure 2). The total synthesis of (±)-puupehenone was firstly described in 1978, when G. L. Trammel showed a method that applied acid-mediated cyclization of sesamol derivatives [12]. Twenty years later, Barrero et al. detected a lack of reproducibility in this method, and they reported the enantiospecific synthesis of (+)-puupehenone from the bicyclic diterpene (−)-sclareol, a fragrant compound extracted from clary sage flowers (*Salvia sclarea*) [13]. The same group proposed an improved method for puupehenone synthesis [14]. However, these are not the only approaches for the synthesis of puupehenone; in 2002, an alternative and shorter synthetic route starting from (+)-sclareolide was described, in which the heterocyclization needed for the synthesis of the molecule was mediated through the presence of an oxygen function at C-8 of (+)-sclareolide [15].

Different strategies targeting the synthesis of the tetracyclic core of puupehenones were further developed, providing new and improved synthetic routes. Thus, Wallace and collaborators described a three-step stereoselective reaction to access the tetracyclic core of puupehenone and 15-oxopuupehenol using methal-free radical cyclisations [16]. In addition, the construction of the tetracyclic core of puupehenone by using the Diels–Alder reaction of 2-ethenyl-1,3,3-trimethylcyclohexene with 4H-chromen-4-ones has been described [17].

In addition to the use of (−)-sclareol as a starting point for the synthesis of puupehenone [13], this easily commercially available compound has been used for the synthesis of other puupehenone-related compounds, as is the case of 15-oxopuupehenol, puupehedione, 15α-cyanopuupehenone [14], 8-epipuupehedione [18] and others [19]. Concerning the synthesis of 8-epipuupehedione, different approaches have also been described. After the report of Álvarez-Manzaneda et al. [18], the same group described the synthesis of this molecule using natural drimenol as the initial molecule [20]. Moreover, 8-epipuupehedione and puupehedione syntheses were previously described through concomitant *O*-allyl deprotection and electrocyclization of an intermediate dione molecule derived from (−)-carvone [21]. In a more recent study, Dixon and collaborators developed a scalable, divergent synthesis of meroterpenoids through the invention of a “borono–sclareolide” precursor that allowed a high-yield of (+)-chromozonarol. This intermediate was used for the subsequent syntheses of a variety of meroterpenoids, including (+)-8-epipuupehedione [22].

The syntheses of (+)-chloropuupehenone, (+)-choloropuupehenol and their stereoisomers were described for the first time by Hua and collaborators. In that report, the authors investigated two synthetic routes to get (+)-chloropuupehenone, trying to improve the final yield of the molecule [23].

## 3. Biological Activities of Puupehenones

Puupehenones have been described as a family of compounds with very diverse and interesting biological effects. Different activities have been reported for a number of puupehenones, including antiangiogenic, antitumoral, antioxidant, antimicrobial, immunomodulatory and antiatherosclerotic effects. Here, we summarize the main information about the biological activities detected for puupehenones.

### 3.1. Antiangiogenic Activity

In a blind screening for the search of potential antiangiogenic compounds, puupehenone was selected due to its efficacy in inhibiting the formation of tubular-like structures on Matrigel in bovine aortic endothelial cells (BAEC) at a very low dose (3 µM) [8]. In addition to puupehenone, 11 structurally-related compounds (all of which were terpenylquinones with a labdane-type decalin ring, either natural products from marine origin or their synthetic derivatives) were evaluated, showing that some of these compounds exhibited even more potent antiangiogenic activity than puupehenone, with inhibitory doses as low as 0.37 µM for some of them. Interestingly, the 12 compounds studied showed a weak effect on BAEC cell growth, with IC_50_ values ranging from 7–45 µM, which imply that the observed effect of these compounds on the tube formation assay was not due to the inhibition of cell growth. In addition, these IC_50_ values did not differ from those obtained in tumor cell lines (human lung carcinoma, colon and pancreatic adenocarcinomas, breast carcinoma and glioblastoma cell lines), demonstrating that the effects of the assayed puupehenones on cell growth were not specific for endothelial cells. In vivo assays on chick chorioallantoic membrane (CAM) showed that puupehenone did not exhibit significant antiangiogenic effect at the assayed conditions, but in contrast, three of the related compounds studied (8-epipuupehedione, 8-epi-9,11-dihydropuupehedione and isozonarol, which is another terpenylquinone with related structure) demonstrated a very potent inhibitory effect on the CAM neovascularization, at doses of 30 nmol/CAM or even lower. Furthermore, zymographic experiments with those three antiangiogenic puupehenone-related compounds showed their activity in inhibiting the production of urokinase-type plasminogen activator (uPA) by endothelial cells. uPA is a secreted serine protease that converts plasminogen, an extracellular matrix protein, into plasmin. Related to this finding, the three selected compounds were able to inhibit the invasive capacity of endothelial cells in vitro in a modified Boyden chamber assay. Additionally, one of them (8-epipuupehedione) interfered in vitro with another important step in the angiogenic process, namely, the migration of endothelial cells. Indeed, 8-epipuupehedione, a synthetic derivative of puupehedione, was the most active compound assayed [8].

Recently, a study focused on the search for novel antiangiogenic scaffolds, pointed again to the potential of puupehenone as an inhibitor of angiogenesis [24]. In that work, 71 natural and semisynthetic compounds were filtered by a bioinformatic system attending to their novelty and druggable functionalities. Using this tool, 38 compounds were selected, tested in angiogenesis in vitro assays and screened in an angiogenesis-targeted biochemical kinase profiling. Puupehenone was one of the resulting hits, showing a high efficacy to inhibit VEGF-mediated endothelial tube-like formation in vitro. Although this compound did not progress further to the kinase profiling secondary assays, virtual screening by molecular docking of puupehenone against a panel of selected angiogenesis-related kinases suggested that glycogen synthase kinase-3 beta (GSK-3β) could be a possible kinase target of the molecule. GSK-3β is a serine/threonine kinase involved in the regulation of different cellular processes. In addition to its role in cell proliferation and inflammation, GSK-3β has been reported to play an important role in angiogenesis by inducing proangiogenic factors. The structural results relating to the binding mode of puupehenone to GSK-3β revealed that chemical modifications in the molecule could improve this binding, which offers an excellent starting point to design puupehenone-based GSK-3β inhibitors [24].

### 3.2. Antitumoral Effects of Puupehenones

First evidence of the antitumoral effect of puupehenone was reported by Kohmoto et al. in 1986 [25]. In that work, some values of IC_50_ for puupehenone in tumor cell lines (murine leukemia, human lung, colon and breast cancer cell lines) were shown [25], although these data were provided as ranges of values that differed in one order of magnitude (from 0.1–1 µg/mL in human lung carcinoma; from 1–10 µg/mL in human colon cancer). After these observations, more precise information about the effect of puupehenones on tumor cell lines has been reported [8,19,26,27]. Indeed, in the above-mentioned work by Castro et al., focused on the potential antiangiogenic activity of puupehenone and structurally related compounds, the authors showed the capacity of these compounds to inhibit the growth of several tumor cell lines. Reported values of IC_50_ ranged from 4 µM to more than 15 µM for the different studied compounds in a panel of cell lines [8].

As shown in Table 1, puupehenones have been tested in different tumor cell lines.

However, little is known about the exact mechanism of action of these compounds to inhibit tumor cell growth. In trying to figure out its mode of action, the in vitro antitumoral activity of 8-epipuupehedione on human promyelocytic leukaemia cells (HL-60) was investigated [9]. In these cells, this compound showed a IC_50_ value lower than those obtained for other tumor and non-tumor cell lines, suggesting a certain specificity in the growth inhibition of leukaemia cells. Indeed, 8-epipuupehedione induced apoptosis in HL-60 leukaemia cells and in bovine aortic endothelial cells (BAEC), producing DNA fragmentation and effector caspase-3 activation, but these effects were not observed in the human colon adenocarcinoma cell line HCT-116. Interestingly, results in that work showed that the induction of apoptosis was stronger in the HL-60 leukaemia cell line than in BAEC. Furthermore, in leukaemia cells, 8-epipuupehedione strongly inhibited the secretion of the extracellular matrix remodeling enzyme metalloproteinase-2 (MMP2) and uPA production. This study demonstrated that 8-epipuupehedione is a potent apoptosis inductor in HL60 leukaemia cells, and a modulator of the extracellular-matrix remodeling capacity of this cell line, suggesting that in addition to its antiangiogenic activity, this compound could display a potential therapeutic effect in the treatment of promyelocytic leukaemia [9].

In a recent report, puupehenone was selected in a cell-based screen to identify natural products that were able to modulate HIF-2α in the context of renal cell carcinoma [28]. Results presented in that work showed that puupehenone inhibited HIF-2α-induced transcription of target genes. Interestingly, the data suggested that this modulatory activity might be selective for HIF-2α vs. HIF-1α. HIF-α transcription factors (HIF-1α, HIF-2α and HIF-3α) are key elements triggering the cellular response to hypoxia. Target genes of HIF encode for proteins involved in important processes, such as angiogenesis, metabolism and cell survival, that allow the cell to survive under low oxygen conditions [29]. Upregulation of the HIF pathway has been reported in several cancer types, either due to intratumoral hypoxia or to genetic mutations, and this feature correlates with a poor prognosis [30]. In renal cancer, HIF-2α has an important role in tumorigenesis [31]. Therefore, the search for compounds that inhibit this factor is an interesting antitumoral approach. This study provides a possible mechanism of action of the antitumoral effect of puupehenone in certain cancer types that rely on HIF-2α to progress, as is the case of renal cancer.

### 3.3. Antioxidant Activity of Puupehenones

One interesting property exhibited by some compounds of the puupehenone family is their antioxidant capacity. Puupehenone showed strong antioxidant activity in both a 2,2-diphenyl-1-picrylhydrazyl radical (DPPH) solution-based chemical assay and a 2′,7′-dichlorodihydrofluorescein diacetate (DCFH-DA) cellular-based assay, demonstrating that this compound has not only an inhibitory effect in a solution-based antioxidant assay but can also be taken up by living cells and maintain its inhibitory activity [32]. Puupehenol has been described as a potent antioxidant metabolite [33]. Isolated from a Hawaiian deep-water *Dactylospongia* sp. sponge, puupehenol and puupehenone exhibited very similar strong antioxidant activities in the ferric reducing antioxidant power (FRAP) assay [34,35].

The exact mechanism of these compounds to exert their antioxidant effect is not well-understood, but interestingly some reports have shown that puupehenone and other related compounds inhibit human lipoxygenases [36,37]. Lipoxygenases (LOX) are a family of enzymes involved in the synthesis of leukotrienes from arachidonic acid, a very important step in the inflammatory process [38]. In addition, the implication of these enzymes in the reactive oxygen species (ROS) formation has been reported [39]. In a screening focused on the search for new lipoxygenase inhibitors, puupehenone and four related compounds (chloropuupehenone, methoxypuupehenone, dimethoxypuupehenol and 20-methoxy-9-,15-ene-puupehenol) were tested as potential inhibitors of 15-LOX and 12-LOX, using an assay that directly measures the product formation of the enzymes by spectrophotometry [36]. In this study, all the five compounds exhibited an inhibitory effect against human 15-LOX, 12-LOX and 15-soybean lipoxygenase; in contrast, their inhibitory activity against 12-LOX was moderate (with IC_50_ of 8.3 µM for puupehenone). Interestingly, puupehenone was the most potent inhibitor of 15-LOX, with an IC_50_ value of 0.76 µM. The most active compound in the inhibition of 12-LOX was chloropuupehenone, with IC_50_ of 0.7 µM. In addition to 15-LOX and 12-LOX, the inhibitory effect of puupehenones against 5-LOX (a lipoxygenase isoform typically involved in inflammatory diseases such as asthma but with an emerging role in cancer [40]) has been studied, showing that puupehenone exhibited a high inhibitory activity against 5-LOX. The selectivity observed for puupehenones in these assays was diverse, but in general these compounds did not exhibit a very high selectivity against the studied lipoxygenases, with the exception of puupehenone, which presented a moderate selectivity for 5-LOX vs. 12-LOX [37].

In an assay using beef heart submitochondrial particles, the potential activity of puupehenone and five related compounds as inhibitors of the integrated electron transfer chain, in particular NADH oxidase (NOX) activity, was tested [41]. NOX enzymes are a family of proteins that transfer electrons across biological membranes. As a consequence of their activity, a superoxide ion is produced, therefore generating ROS [42]. In the work by Ciavatta et al., all the six puupehenone-related compounds assayed showed an inhibitory effect against NOX activity, with IC_50_ values that ranged from 1.3 µM (for puupehenone, the most potent inhibitor of NOX activity in this study) to 44 µM (for bispuupehenone) [41].

Altogether, these observations could partially explain the antioxidant activity of puupehenones in cells by a putative mechanism that involves lipoxygenases and NOX inhibition.

### 3.4. Antimicrobial Activities of Puupehenones

Since puupehenone was first isolated and described as an active compound against Gram-positive bacteria and some fungi strains [4], several studies have reported antimicrobial activity for puupehenones (including antibacterial, antifungal, antiviral and antimalarial activities). Hamann and collaborators described, in 1993, the antifungal activity of puupehenone, cyanopuupehenol, puupehedione and chloropuupehenone against *Aspergillus oryzae*, *Penicillium notatum*, *Trichophyton mentagrophytes*, *Saccharomyces cerevisiae* and *Candida albicans* [7]. In [41], puupehenone and five related compounds were tested for antifungal and antibacterial activities. In that work, puupehenone showed moderate activity against *Candida albicans* and *Staphylococcus aureus*. Similar antimicrobial activities against *S. aureus* and the fungus *Candida tropicalis* have been reported for 15α-methoxypuupehenol [27]. A potent antifungal activity for puupehenone has been reported against *Cryptococcus neoformans* and *Candida krusei* [43]. The growth of the Gram-positive bacteria *S. aureus* and *Bacillus cereus* is also inhibited by puupehenol, showing an inhibitory activity very similar to puupehenone [33].

The antituberculosis activity of puupehenones has been reported [44]. At a concentration of 12.5 µg/mL, puupehenone, 15α-cyanopuupehenol and 15-cyanopuupehenone exhibited 99%, 96% and 90% inhibition against *Mycobacterium tuberculosis*, respectively [7]. In a recent study, two puupehenone derivatives, namely, 15α-methoxypuupehenol and puupehedione, showed similar activity against *M. tuberculosis* as that previously reported for puupehenone. Interestingly, both compounds had high selectivity against dormant bacteria, which is a specific non-replicative status of the microorganism that renders a phenotype tolerant to front-line drugs during infection [45].

Puupehenones exhibit antiviral activity. Cyanopuupehenone and puupehedione showed potent antiviral activity in different infection models (more than 80% reduction in cell infection) [7]. In addition, bispuupehenone and 15-oxopuupehenol have been reported to produce moderate reduction of viral infection [46].

Interestingly, puupehenone, 15α-methoxypuupehenol and 15-oxopuupehenol have been reported to exhibit an antimalarial effect, with low IC_50_ values against different strains of *Plasmodium falciparum* [27,46].

### 3.5. Immunomodulatory Activity of Puupehenones

Using the mixed lymphocyte reaction (MLR) test [47], 15-oxopuupehenol, cyanopuupehenol, cyanopuupehenone, puupehenone, 21-chloropuupehenone, puupehedione and dipuupehetriol have been shown to modulate the immunological response of T cells in vitro [7,46]. In these experiments, puupehedione was the most active compound. Little is known about the immunomodulatory role of puupehenenones, since to our knowledge there has been no further research into this remarkable activity in the literature. In an interesting study focused on the use of natural products to modify covalent biomolecules that are involved in the modulation of cellular immune responses, puupehenone was attached onto [Leu^27^]MART-1_26-35_, a modified HLA-A2-associated decapeptide identified to function as an epitope for melanoma-reactive cytotoxic T lymphocytes [48]. In spite of the low affinity of the generated adduct for the HLA-A2 molecules, it was able to moderately activate interferon-γ (IFN-γ secretion in peripheral blood and tumor-infiltrating lymphocyte clones.

### 3.6. Puupehenones and Atherosclerosis

Recently, a potential role of puupehenones targeting atherosclerotic disease has been reported [49]. Atherosclerosis is a cardiovascular disease caused by the formation of atheroma lesions in the vessel walls of large and medium arteries. The inflammatory response is chronically activated in atherosclerosis. During the progression of the disease, atheroma lesions accumulate lipids and cholesterol transported by circulating low-density lipoprotein (LDL), but in contrast, high-density lipoprotein (HDL) can affect reverse cholesterol transport, transferring cholesterol from the lesions to the liver for its excretion [50]. In the work of Wahab et al., 19-methoxy-9,15-ene-puupehenol and 20-methoxy-9,15-ene-puupehenol have been reported to up-regulate the activity of the scavenger receptor class B Type-1 (SR-B1, a plasma membrane receptor for HDL that mediates cholesterol transfer to and from HDL) in a SR-B1 stably expressing model of a human hepatocarcinoma cell line. Due to their high efficacy, these two compounds could be considered as full agonists of the receptor, pointing to their potential effect in the reduction of atherosclerosis progression [49].

## 4. Final Remarks and Future Challenges

**Figure 3:**
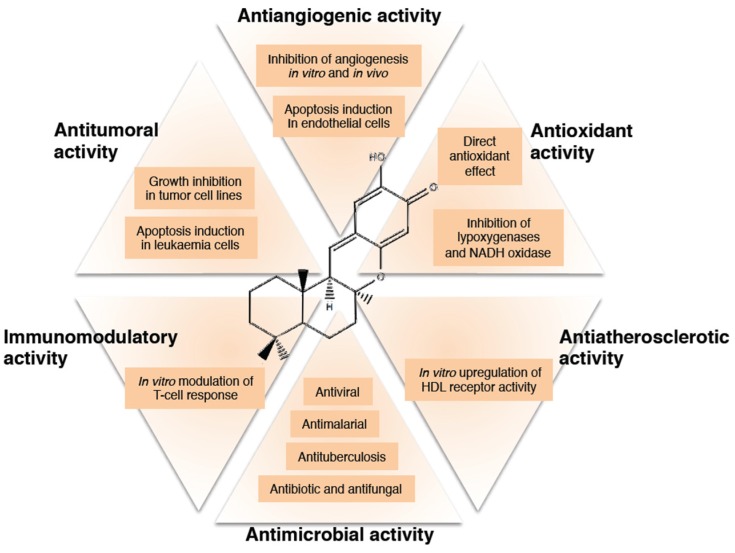
A summary of the multiple bio-active effects of puupehenone and derived compounds with potential therapeutic interest.

Although the number of reports found in the literature about puupehenones is not very large, the high diversity of compounds belonging to this family and the versatile and interesting biological activities reported for them (Figure 3) make puupehenones an excellent target for biomedical research.

From a chemical point of view, the unique characteristics of puupehenones’ structure provide an excellent scaffold for the rationale design of therapeutic agents that could improve the treatment of current resistance-associated human diseases, such as cancer. This feature has been indeed put into use for the search of new antiangiogenic and kinase inhibitor compounds [24].

Biological activities detected for puupehenones are very diverse, and their antitumoral role represents one of the most interesting effects in biomedical research. There are, however, no reports about the systemic effect of these compounds in in vivo cancer animal models, which could improve the knowledge of the antitumoral potential of puupehenones. Taking into account the antiangiogenic activity of some puupehenones [8], namely, the reported pro-apoptotic effect of 8-epipuupehedione in endothelial and leukaemia cells [9], and the inhibitory activity of puupehenone on the HIF-2α transcriptional response [28], these compounds represent a very promising putative drug against cancer. However, the exact mechanism of action of puupehenones in tumor cell lines has not been figured out yet, which opens an opportunity for future research.

Apart from their antitumoral role, the inhibitory activity of puupehenones on lipoxygeneses deserves the attention of future investigations [36,37]. In addition to ROS generation, lipoxygenases are implicated in inflammatory diseases since these enzymes catalyze the formation of eicosanoids (prostaglandins and leucotrienes) from polyunsaturated fatty acids such as linoleic and arachidonic acids [38]. Once again, the lack of in vivo data in animal models treated with puupehenones is a point to solve in subsequent studies. The same rationale could be applied to the very recent finding of the putative inhibitory role of puupehenones in atherosclerosis, a highly prevalent inflammatory disease [49]. In vivo experiments in atherosclerosis mice models, such as apolipoprotein-E knockout mice (*ApoE^−/−^*) would shed some light on this promising therapeutic application of puupehenones.

Another important finding about puupehenones is their potential in the modulation of immune responses in T cells in vitro. This activity has been reported [7,46], but further research is needed to fully understand the molecular basis of this interesting effect.

In summary, the sponge-isolated compounds puupehenones and their synthetic derivatives represent an open field of investigation for biomedical and pharmaceutical research, and deserve the close attention of the scientific community. The lack of in vivo data about the different effects of puupehenones in several diseases could be the principal goal of future research projects on this issue, since this information could shed some light on the putative use of puupehenones as therapeutic agents.

## Figures and Tables

**Table 1:** IC_50_ values of puupehenones in different cell lines.

COMPOUNDS	TESTED CELL LINES												
	A549	HT29	KB	CV1	MEL28	H116	PSN1	SKBR3	T98G	HCT8	MCF7	P388	BAEC
**Puupehenone**	0.4; 0.5; 0.1–1; **7**	0.2; 0.5	0.5	0.5	N.D.	**8**	**5**	**15 µM**	**>15**	1–10	0.1–1	1.3; 0.25; 1	**10 ± 2**
**Bispuupehenone**	N.D.	N.D.	N.D.	N.D.	N.D.	N.D.	N.D.	N.D.	N.D.	N.D.	N.D.	N.D.	N.D.
**15-oxopuupehenol**	N.D.	N.D.	N.D.	N.D.	N.D.	N.D.	N.D.	N.D.	N.D.	N.D.	N.D.	N.D.	N.D.
**Puupehedione**	1–2	1–2	N.D.	N.D.	1	N.D.	N.D.	N.D.	N.D.	N.D.	N.D.	1	N.D.
**Puupehediol**	2.5–> **15**	2.5	N.D.	N.D.	2.5	**>15**	**>15**	**>15**	**>15**	N.D.	N.D.	1	**27 ± 2**
**Cyanopuupehenol**	2	2.5; 2	N.D.	N.D.	2	N.D.	N.D.	N.D.	N.D.	N.D.	N.D.	2	N.D.
**8-epipuupehedione**	0.25–> **15**	0.25	N.D.	N.D.	0.25	**>15**	**>15**	**>15**	**>15**	N.D.	N.D.	0.25	**28 ± 6**
**8-epi-9-dihydropuupehedione**	5–> 15	5	N.D.	N.D.	5	**>15**	**>15**	**>15**	**>15**	N.D.	N.D.	5	**35 ± 7**
**8-epipuupehenol**	1.2	1.2	N.D.	N.D.	1.2	N.D.	N.D.	N.D.	N.D.	N.D.	N.D.	1.2	N.D.
**Cyanopuupehenone**	5–> **15**	1–2.5	N.D.	5	N.D.	**>15**	**>15**	**>15**	**>15**	N.D.	N.D.	5	**11 ± 1**
**21-chloropuupehenone**	0.5	0.5	N.D.	0.5	N.D.	N.D.	N.D.	N.D.	N.D.	N.D.	N.D.	0.2	N.D.
**Dipuupehetriol**	1	10	N.D.	0.25	N.D.	N.D.	N.D.	N.D.	N.D.	N.D.	N.D.	5	N.D.
**15a-methoxypuupehenol**	N.D.	N.D.	6	N.D.	N.D.	N.D.	N.D.	N.D.	N.D.	N.D.	N.D.	N.D.	N.D.
**8-epipuupehediol**	**9 ± 1**	N.D.	N.D.	N.D.	N.D.	**10 ± 1**	**1 ± 4**	**>15**	**>15**	N.D.	N.D.	N.D.	**27 ± 5**
**8-epi-9,11-dihydropuupehediol**	**>15**	N.D.	N.D.	N.D.	N.D.	**12 ± 2**	**11 ± 4**	**10 ± 5**	**>15**	N.D.	N.D.	N.D.	**17 ± 2**
**Acetylpuupehenone**	**8 ± 4**	N.D.	N.D.	N.D.	N.D.	**8 ± 4**	**>15**	**10 ± 3**	**>15**	N.D.	N.D.	N.D.	**7 ± 1**

IC_50_ values are expressed in µg/mL, unless values in bold in the table, which correspond to µM. A549, human lung carcinoma; HT29, human colon adenocarcinoma; KB, human cervix carcinoma; CV1, monkey kidney fibroblasts; MEL28, human melanoma; H116, human colon adenocarcinoma; PSN1, human pancreatic adenocarcinoma; SKBR3, human breast carcinoma; T98G, human glioblastoma; HCT8, human colon cancer; MCF7, human breast cancer; P388, mouse leukaemia; BAEC, bovine aortic endothelium. Data compiled from [7,8,19,25,26,27].
